# Eye Safety Related to Near Infrared Radiation Exposure to Biometric Devices

**DOI:** 10.1100/tsw.2011.52

**Published:** 2011-03-01

**Authors:** Nikolaos Kourkoumelis, Margaret Tzaphlidou

**Affiliations:** Department of Medical Physics, Medical School, University of Ioannina, Ioannina, Greece

**Keywords:** infrared radiation, safety, eye, retina, nonionizing radiation, biometrics

## Abstract

Biometrics has become an emerging field of technology due to its intrinsic security features concerning the identification of individuals by means of measurable biological characteristics. Two of the most promising biometric modalities are iris and retina recognition, which primarily use nonionizing radiation in the infrared region. Illumination of the eye is achieved by infrared light emitting diodes (LEDs). Even if few LED sources are capable of causing direct eye damage as they emit incoherent light, there is a growing concern about the possible use of LED arrays that might pose a potential threat. Exposure to intense coherent infrared radiation has been proven to have significant effects on living tissues. The purpose of this study is to explore the biological effects arising from exposing the eye to near infrared radiation with reference to international legislation.

## INTRODUCTION

Biometric methodologies manipulate a measurable biological characteristic of the human anatomy. For a successful measurement, the quality of many parameters, such as universality, uniqueness, permanence, collectability, accuracy, and acceptability or medical safety, must be evaluated in advance. In particular, medical implications can be divided into two classes: indirect (IMI) and direct (DMI). IMI concern information disclosure from a medical database without authorization, while DMI signify a potential threat due to biological effects.

The biometric systems that analyze the complex and distinctive characteristics of the eye can be divided into two categories: iris biometrics and retina biometrics. Iris recognition analyzes the random textured patterns of the iris by illuminating near infrared (NIR) radiation[1]. Compared to visible, NIR light offers superior resolution due to its low absorption by the principal iris pigment melanin and, consequently, it allows the structural patterns of the iris to be imaged with greater contrast. Retinal scanning is accomplished by illuminating the retina with a low-intensity NIR and imaging the patterns formed by the blood vessel network.

The objective of this study is to review eye safety issues in relation to infrared radiation utilized by many biometric devices.

## INFRARED RADIATION

The infrared region exists between the visible and microwave subdivisions of the electromagnetic spectrum and is divided into the following three, biologically significant, bands: IR-A (near IR) between 760 and 1400 nm, IR-B (mid IR) between 1400 and 3000 nm, and IR-C (far IR) between 3000 and $10^6$ nm[2]. Current infrared biometric methodologies rely on IR-A radiation. In general, infrared radiation is classified as nonionizing radiation with quantum energy <12 eV. Although its energy is deficient for ionizations, it may cause diverse biological effects of thermal origin. In particular, infrared radiation induces molecular vibrations and rotations, and principally interacts with tissues that generate heat. It is unperceived by any of the human senses, unless from a heat stimulus resulting from high intensity. Specifically, intense IR-A exposure of the eye may cause retinal burns and cataractogenesis[3,4]. The iris absorbs between 53 and 98% of the infrared light within a spectrum of 750 to 900 nm[5], but the degree of pigmentation affects the absorption's percentage[6]. The crystalline lens transmits most wavelengths up to 1400 nm, while a fraction of NIR absorption happens above 900 nm. IR-A does not interfere significantly with the vitreous and aqueous body since its absorption properties resemble more to water involving IR-B and IR-C. The extent of biological effects from nonionizing radiation exposure is based mainly on the energy of the incident radiation, the power density of the field or beam, the duration of exposure, the spatial orientation, and the biological characteristics of the irradiated tissues, such as molecular composition, blood flow, pigmentation, and functional importance[7].

## EYE DAMAGE MECHANISM OF IR-A RADIATION

Generally, wavelengths between 760 and 1400 nm are invisible to the cornea by 96%; they are transmitted through the ocular media and focus on the retina (Fig. 1). As noted, in this spectral region, thermal effects dominate and extreme exposure of the retina and the choroid causes enzyme denaturation due to critical temperature rise. Regenerative capabilities of the retina are very limited and, therefore, any damage results in severe loss of visual acuteness.

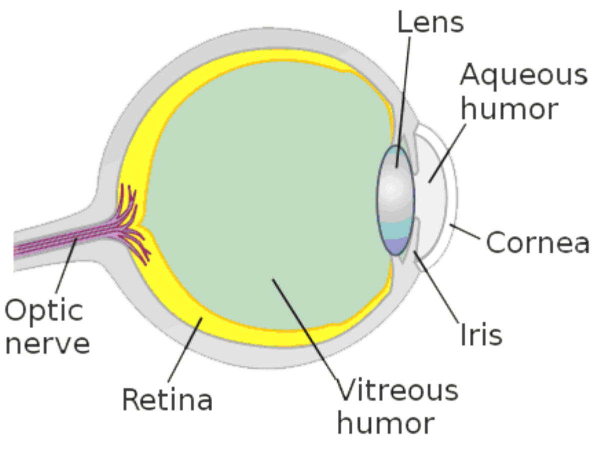

**FIGURE 1**. The cross-section of the human eye.

Thermal injury is a zero-order rate process[8], and depends on energy absorption and scattering of the infrared light in a volume of tissue for a given amount of incident energy delivered to a tissue by a light source[9]. Scattering stems from the inhomogeneous composition of the tissue exhibiting variations in the refraction index, while absorption originates from the type of the light propagation media. In the case of the retina, the absorbing NIR chromophore is melanin, which is concentrated in the retinal pigment epithelial (RPE) cells and focally in the choroid[10]. The optical penetration depth, $\delta$, is determined by the simultaneous action of the absorption, scattering, and anisotropy of the tissue, and corresponds to the distance at which the fluence rate is reduced to exp(-1) of the initial value. It is evaluated as[6]:

$$\delta = \frac{1}{\sqrt{3\mu_\alpha(\mu_\alpha + \mu_s')}} \tag{1}$$

where $\mu_s'$ is the reduced scattering coefficient and $\mu_\alpha$ the absorption coefficient of the medium; both are dependent on the wavelength of the incident radiation.

Retinal irradiance (exposure rate, $E_r$) is the crucial parameter for the evaluation of thermal damage and is directly correlated to source radiance (brightness, $L_s$). It is computed as the radiant power ($P$) divided by the retinal focus area and is usually expressed in Wcm$^{-2}$[11]:

$$E_r = \frac{\Delta P}{\Delta A'} = \frac{\tau \int_A \frac{\Delta A}{f^2} L_s dA}{\Delta A'} \tag{2}$$

where $f$ is the effective focal length of the eye, $\Delta A$ the area segment of the anterior, which is conjugated to the retinal area segment $\Delta A'$, and $\tau$ is the transmittance of the ocular media. Based on the Gullstrand-LeGrand model for the adult human eye, the focal length equals 1.7 cm and the above equation simplifies to[12]:

$$E_r = 0.27 \cdot \tau \cdot L_s \cdot d_p^{\,2} \tag{3}$$

where $d_p$ is the pupil diameter.

Eq. 3 shows that the retinal irradiance is dependent on the accommodation and the age of the eye as a function of pupil aperture. The energy flux on the retina increases as the diameter of the pupil increases since the size of the image on the retina varies inversely with the pupil diameter.

Retinal irradiance is much higher than the corresponding irradiance at the cornea or lens since light is focused to a small area by the physical converging lens. This effect is even greater for the case of NIR due to the crystalline transparency to this wavelength. The equation that relates the irradiance of the cornea ($E_c$) with the corresponding irradiance on the retina is[13]:

$$E_r = E_c \left( \frac{d_p}{d_r} \right)^2 \tag{4}$$

which takes into account the pupil diameter diaphragm and the retinal spot diameter, $d_r$. According to this, the irradiance at the retina can be $10^6$ times more intense than that on the cornea based on a pupil diameter of 7 mm, which is regarded as the upper limit of the iris aperture forming a retinal image between 10 and 20 μm. For coherent optical sources (laser), energy deposition is virtually independent of the distance. It must be strongly emphasized, however, that none of the current eye biometric devices use collimated beams; instead, they utilize incoherent illumination that is produced by light emitting diodes (LEDs, or IREDs in the infrared spectrum region). This radiance power is $10^3$ times lower than that of a laser, using a rough approximation[14]. When the eye approaches a LED-type diverging beam at distances greater than the shortest focal length, the potential risk increases. In contrast, for distances less than the shortest focal length, the energy concentration is decreased due to the increase of the retinal illumination area[15].

Thermal damage does not obey the Bunsen–Roscoe law[16] and is heavily affected by the conduction of heat away from the irradiated tissue. The light damage mechanism in relation to thermal injury adopts the critical concept of thermal confinement[17]. The latter describes the rate of energy deposition and the rate of heat dissipation of the irradiation area. Thermal confinement occurs when radiant energy changes the equilibrium, producing thermal injuries. Under this viewpoint, the equilibrium of thermal energy of a tissue volume can be maintained when the heat gained by the irradiance and from the neighboring conductive volumes is lost, through either boundary tissues (or via blood flow) or the work done by the tissue. On the whole, 45$^{o}$C is the lower limit for thermal injury, but the critical temperature depends on duration and radiant power[18]. Irradiation area and ambient temperature are two additional factors that play a major role in tissue cooling since this is performed through conduction. Sudden temperature elevations of the anterior of the eye due to high-level infrared exposure are readily sensed, causing pain to motivate the eye to move or blink in order to restrict the duration of exposure. Nevertheless, IR-A light initiates a very low visual stimulus and, for this reason, the aversion response that typically shields the eye from excessive continuous irradiation of 0.25 sec or more is not activated. This is the main reason for considering the pupil of the eye completely dilated and eyelids fully opened as a prerequisite for calculating exposure limits.

The etiology of cataractogenesis induced by infrared radiation is not clearly understood, although it is known that cataract provoked under chronic low-level exposure requires many years to be formed. In contrast, this is not true for laboratory animals exposed to a high irradiation dose[19]. There were two contradictory theories stating that cataract formation is realized either by heating of the lens by direct absorption[20] or through conduction of heat from the epithelium[21]. Experimental work on monkey eyes by Pitts and Cullen[22] showed that both effects act synergistically since the iris, being a highly vascular tissue, easily absorbs heat, which potentially denatures the lens' proteins aided by the increase in temperature in the anterior eye and eventually contributes to posterior opacities.

## THERMAL MODELING

The simulation of the temperature distribution in the eye under local heating is an essential feature of the construction of infrared and radiofrequency safety guidelines. To predict ocular damage, mathematical models of the eye are employed since the *in vivo* temperature measurement of the interior of the human eye is not possible. Within the scope of these models, properties that must be taken into consideration are of anatomical, physiological, and refractive type. Generally, the transport of thermal energy in living tissue is a highly complex procedure involving multiple mechanisms, including conduction, convection, radiation, metabolism, evaporation, and phase change[23]. Temperature distribution in the human eye is simulated by using finite element methods (FEM)[24,25,26] and finite difference methods (FDM)[27,28]. Okuno[28] has suggested that the threshold irradiance for cataract formation has a direct dependency on the temperature rise and an inverse one on the temperature rise of the lens for the incident irradiance. In general, this varies with exposure time, which must be >30 sec in order to establish a safety threshold irradiance limit of, approximately, 80 $mWcm^{-2}$. Lagendijk[29] also applied FDM to calculate the temperature distribution in human and rabbit eyes during hyperthermic treatment of tumors, proving that the thermal conductivity of the lens is much lower than that of water. Hirata et al.[30] also used FDM to study temperature variations in the human eye exposed to electromagnetic waves. Nevertheless, FEM are superior since the model grids are not rigid and, therefore, they can mimic the ocular surface more precisely[31]. Temperature rises in the human eye induced by infrared radiation was extensively studied by Scott using FEM[32]. In this mathematical model, the eye is divided into six isotropic and homogeneous subdomains. Using some simplifying assumptions concerning the geometry and structure of the eye, the governing differential equation for the temperature distribution is the bioheat transfer equation in the interior of the eyeball[33,34]:

$$\rho c \frac{\partial T}{\partial t} = \nabla(k \nabla T) + H \quad (5)$$

where $\nabla T$ is the gradient (maximum change unit distance) of the temperature $T$; $\rho$, density; $c$, specific heat capacity; $\rho c$, volumetric specific heat; $k$, thermal conductivity; $t$, time; and $H$, internal heat source when the eye is exposed to infrared radiation. $H$ includes factors such as the pupil size, the eyelid shielding, the image size, the transmission properties of the ocular media (see Eq. 1), the minimum and maximum wavelengths of the radiation emitted by the source, together with the source temperature and the irradiance incident on the eye[32]. For this study, we must consider transient temperature variations as the relevant biometric systems expose the human eye intermittently. Based on this model, an exposure of 30 sec under 5000 $Wm^{-2}$ results in a temperature elevation of 1–3$^{o}$C for the posterior surface of the iris and anterior-posterior poles of the lens, while the retina holds an almost steady temperature profile. Approximately 20 min are enough to conduct heat from the anterior tissues to the interior of the eyeball. Furthermore, the application of this model showed that evaporation in the anterior part of the eye and rapid blinking have a considerable influence on lens temperature. Nonetheless, according to recent calculations of heat transfer coefficients, temperature rise is probably even lower[35]. Other researchers[36] have included more parameters and terms, such as blood perfusion and metabolic heat generation, in Eq. 5 in an effort to represent more accurately the concept of thermal confinement than is described above, although not specifically for infrared exposure. Body temperature does not seem to interfere with thermal damage due to radiation exposure and this is also true for wavelengths below the infrared region[37], although it affects temperature discrepancies in the human eye. Another model based on bioheat Eq. 5 was developed by Clark et al.[38], where it was considered that pigment epithelium and the choroid act as uniform absorbers, but with different absorption coefficients. This simple model assumed a uniform beam cross-section and was compared with laboratory experiments on rabbits. Thermal damage depends severely on beam collimation and power density. The process is slow, in contrast to the timescale for photochemical damage produced by different IR-A wavelengths. The profound results produced with well-collimated sources have been described by mathematical models of laser-induced eye lesions[39,40].

## EXPOSURE LIMITS

The International Commission of Non-Ionizing Radiation Protection (ICNIRP) takes for granted that infrared radiation poses a risk to the human eye under certain conditions[41]. The norm regarding thermal lesions for cornea and lens states that the ocular exposure should not exceed 10 $mWcm^{-2}$ for lengthy exposures (>1000 sec) and 1.8 $t^{-3/4}$ $Wcm^{-2}$ for shorter exposure durations. Retinal exposure limit values (ELV) for IR-A irradiation and time beyond 10 sec stem from the following formula, which defines the burn hazard effective radiance when a strong visual stimulus is absent:

$$\sum L_\lambda \times R(\lambda) \times \Delta\lambda \le 0.6/\alpha \quad [Wcm^{-2}sr^{-1}] \quad (6)$$

where $L_\lambda$ is the average measured spectral radiance ($Wm^{-2}nm^{-1}$); $R(\lambda)$, the retinal thermal hazard weighted function; $\Delta\lambda$, wavelength interval; and $\alpha$, angular subtense of the source (radians).

For shorter exposures (10 μsec to 10 sec), the effective radiance associated with the retinal thermal hazard is defined as:

$$\sum L_\lambda \times R(\lambda) \times \Delta\lambda \le 5/\alpha t^{0.25} \quad [Wcm^{-2}sr^{-1}] \quad (7)$$

The angular subtense appears in Eqs. 6 and 7 since retinal image size is proportional to its value. Generally, it is different than the beam spread angle, has a maximum value of 0.1 radians and a minimum effective value dependent on the exposure time (0.25–10 sec). With increasing exposure time, the image is stretched on the retina due to eye and body movement (Fig. 2). In this instance, the source spectral radiance, $L_\lambda$, is calculated using the effectively larger field of view solid angle, which also varies with time, having a value of 0.011 rad for approximately 10-sec exposure.

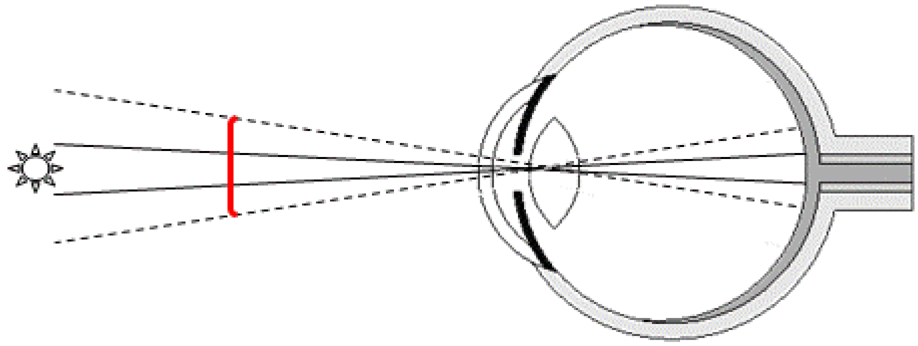

**FIGURE 2**. Simplified diagram showing the lengthened field of view.

The retinal thermal hazard weighted function is wavelength-dependent up to 1050 nm and decays for increasing wavelengths, while from 1200 to 1400 nm, it is even lower and constant (Fig. 3).

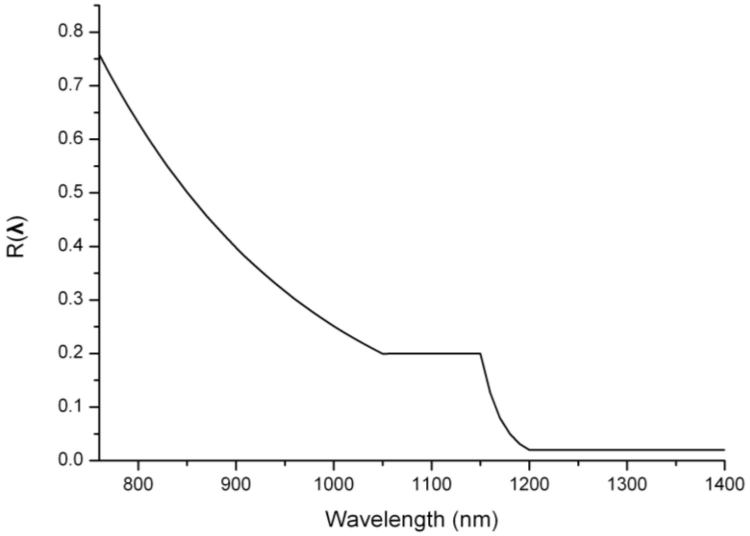

**FIGURE 3**. The thermal hazard action spectrum, $R(\lambda)$, for IR-A wavelengths.

Irradiance is measured at a distance of about 20 cm from the LEDs[14] since this is the lower value for successful eye accommodation and is usually taken as a worst case scenario. The correlation of the radiance exposure limits and irradiation time is depicted in Fig. 4.

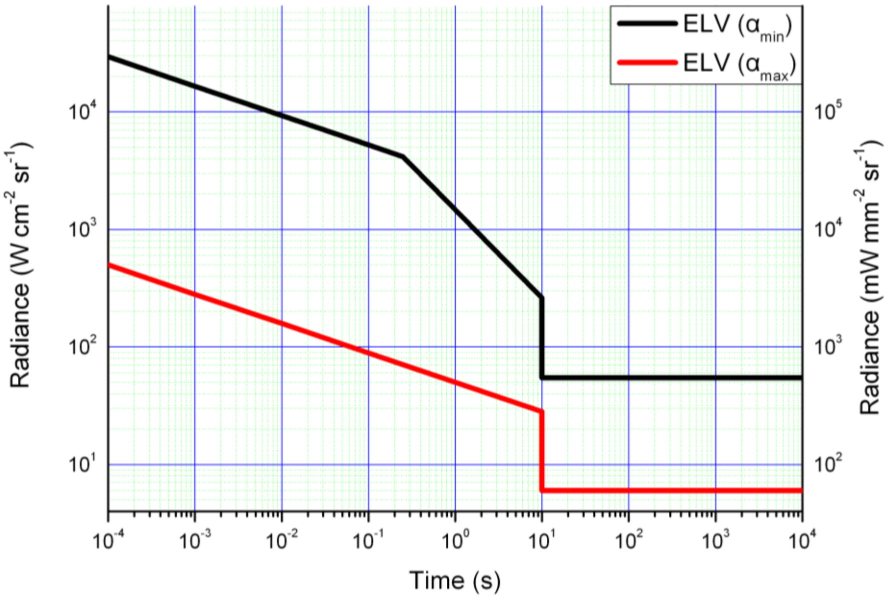

**FIGURE 4**. Retinal ELV for IR-A irradiation as a function of time.

## LEGISLATION – DIRECTIVES FOR INCOHERENT SOURCES

The typical emitting range of IREDs is from 800 to 960 nm. Until 2006, both LEDs and lasers were classified according to EN 60825-1, but this standard overestimated the potential hazards of LEDs since they were classified as laser-type devices. Currently, in Europe, the safety classification of noncoherent (broadband) sources is defined in EN 62471:2008 ("Photobiological Safety of Lamps and Lamp Systems"), which is based on the maximum accessible emission of a product during operation at any time after manufacture. This European norm is derived from CIE S009:2002, published by the CIE (International Commission on Illumination) and afterwards adopted by the IEC (International Electrotechnical Commission) in IEC 62471:2006 and defines a classification scheme based on the time that radiation exposure induces no harm. The scheme involves four risk groups: exempt, with no biological hazard; 1, low risk; 2, moderate risk; and 3, signifying high potential hazard even for momentary exposures. In irradiance measurements, the exposure level at a specific distance can be divided by ELV in order to calculate the hazard value adopting a risk group.

Eye safety is not compromised with a single LED source using today's LED technology, having a maximum radiance of approximately 12 $Wcm^{-2}sr^{-1}$[12]. Multiple LED illuminators, however, may potentially induce eye damage if not carefully designed and used. Biometric devices typically use arrays of LEDs for better results and retinal thermal hazard should be evaluated by taking into account the emitters as a summative source of radiation.

## CONCLUSIONS

With the power of LED illumination increasing exponentially, current legislation must be strictly obeyed to protect the delicate ocular tissues. Although infrared radiation raises the overall temperature of the aqueous eye, affecting mostly the cornea and the aqueous humor[36], IR-A radiation specifically is absorbed by the retina and is very ineffective in producing retinal injuries[42]. Moreover, the procedure of capturing an image suitable for the iris recognition algorithms is very short and can be normally attained within 2–10 sec[43]. LEDs, being normally Lambertian emitters, are significantly different from collimated laser devices, albeit they can be classified into risk groups due to their high intensity. Nevertheless, in the current biometrics industry, safety standards can be possibly overlooked. Thus, academia and industry should ensure that an authentic certification is available to every user before or during the biometric transaction, describing in detail the safety levels of the methodology, adopting a clear statement on the safety of continuous exposure duration. Mathematical modeling of the thermal effects induced by infrared radiation may also provide a suitable simulation environment for *in silico* studies. Epidemiological studies might also be necessary for long-term infrared exposure effects.

Biometric technology is relatively new, but very solid due to technological progress; medical issues, however, have to be addressed successfully prior to public use. Manufacturers of biometric devices must be certain that the rules of international organizations are readily fulfilled and technical data sheets are available for assessment by the scientific community. The latter's responsibility is to improve guidelines and safety standards.
